# Residents’ perceptions of household food waste during the COVID-19 outbreak in Korea

**DOI:** 10.1016/j.heliyon.2022.e11439

**Published:** 2022-11-11

**Authors:** Mona Chang, Walimuni Arachchilage C. S. M, Wisurumuni Arachchilage Hasitha Maduranga Karunarathne, Min-cheol Kim

**Affiliations:** a BK 21 Social Data Science Educational Research Group, Jeju National University, Jeju, 63243, South Korea; b Faculty of Data Science for Sustainable Growth, Jeju National University, Jeju, 63243, South Korea; c Department of Marine Life Science, Jeju National University, Jeju, 63243, Republic of Korea; d Department of Biosystems Technology, Faculty of Technology, University of Ruhuna, Matara, Sri Lanka

**Keywords:** Food waste, Theory of planned behavior, Price consciousness, Risk concerns, COVID-19, Socio-demographic characteristics

## Abstract

Analyzing household food waste data at the global or national level remains a challenge, especially owing to lack of statistical systems and socio-cultural differences. This study determined the factors affecting the intention of households to reduce food waste on Jeju Island and on the Korean mainland. Socio-demographic factors significantly influence household food waste generation. Therefore, studies are often conducted depending on data availability in the corresponding regions. Based on national data and the theory of planned behavior, this study analyzed data using PLS-SEM (Partial Least Squares Structural Equation Modeling) to test the influence of multiple determinants and parameters on dependent variables and investigated the awareness of household food waste in Korea, focusing on Jeju Province, Korea’s largest tourist destination. A survey of 508 local residents established that all factors evaluated in this study, except for risk concerns due to COVID-19, were statistically significant. Among the three antecedents of age, income, and family size, age significantly affected all mediators, directly affecting behavioral intentions. The results are consistent with those of preceding research on the effects of socio-demographic drivers on household food waste generation. The results also indicate that in Korea, where the COVID-19 infection level is lower than that in other countries, residents did not change their food purchasing and waste production patterns. However, a multi-group analysis revealed that the risk concerns caused by COVID-19 differed between residents of Jeju Island and mainland Korea. Overcoming the vulnerability of waste management, including food dumping, is mandatory for locals and tourists on Jeju Island.

## 1 Introduction

The United Nations Environment Program (UNEP) began issuing biannual index reports of global food waste from 2021 onward as part of its plan to establish a food waste scale as one of the 303 indicators of the 17 Sustainable Development Goals (SDGs) [1]. The redistribution of food resources has become an increasingly pressing issue for the equitable survival of the world population, which has been increasing rapidly over the past few decades [2, 3, 4]. UNEP announced that the present volume of food waste is estimated to be approximately 931 million tons annually. Over 60% of which is generated by household consumers [1] according to a report by the Food and Agriculture Organization of the United Nations (FAO), between 2000 and 2017 [5].

Households have been identified as the primary contributors to food waste generation in the supply chain [6, 7, 8]. Households in higher-income countries display a strong tendency to produce food waste [5, 9] because they frequently buy or cook more food than they can consume [8, 9, 10]. For instance, annual food waste generated by an individual varies among countries, from as high as 338 kg in the Kingdom of Saudi Arabia in 2019 to as low as 7 kg in South Africa in 2018 [11]. By applying the life cycle assessment, which assesses the environmental impacts of food waste [12, 13], Skaf et al. [11] found that significant differences in food waste generation across countries are proportional to their impact on climate change.

However, collecting quantitative data from each country is a significant challenge [14, 15, 16, 17] because of the lack of comprehensive knowledge on the quantification of food waste generation and methodologies related to its composition [17, 18, 19] and the complexity of actors at different stages of the food supply chain [20, 21]. Therefore, data that classifies the type of food waste in each region and quantitative food waste emissions data by country presented by research institutes often differ [16, 22, 23]. This hinders the data collection process and its reliability.

Even within the same country, there are differences in the amount of food waste emitted by households depending on the methodology used. A 2018 survey revealed that approximately 63 % of Korean households generated less than 500 g of food waste daily, equivalent to 180 kg annually [24]. However, the Ministry of Environment of Korea reported that in 2017, an average Korean citizen produced 1.02 kg of daily household food waste, amounting for 370 kg annually [25]. In addition to conflicts over the collection procedure and existence of regional data, food waste has become a social controversy because the resources produced for consumption are disposed of before they are completely consumed. Simultaneously, starvation creates conflicts [26] while concern about unwanted food waste and its serious impacts on earth [7] have increased. Although the problem of food waste has been researched and analyzed from economic and environmental perspectives [13, 19, 27, 28], detecting the causes and solutions from a sociological perspective is necessary to understand food consumption and disposal holistically.

Numerous factors that contribute to food waste generation cannot be presented as standard provisions or norms due to regional differences and social, cultural, and demographic backgrounds [3, 17, 19]. As a result, it is clear that sociologists in both developing and developed countries are responsible for establishing a consensus on food waste reduction by understanding general household members' perceptions and behavioral intentions as well as the influential local generators of food waste.

The socioeconomic challenges faced by the people of countries due to COVID-19 lockdowns have made them focus on inevitable issues such as food hoarding, the shelf life of food, and restrictions on movement [29, 30, 31]. Also, an increase in the amount of waste caused by changes in living patterns and the challenges of disposal should also be considered. Therefore, we examined the factors that affect the degree of food waste reduction behavior of local residents in South Korea, including Jeju Province to test the influence of multiple determinants on dependent variables including the public's concern due to COVID-19. Since the effect of COVID-19 on food waste has emerged as a social issue in many aspects, it is expected that timely implications can be derived from this research.

The remainder of this paper is organized as follows: section 2 presents the theoretical literature for the conceptual framework of the research model and a review of food waste including extended timely factors; sections 3 and 4 present the development of research design, methodology, hypotheses, and data analysis based on the survey questionnaires (Appendix). Finally, section 5 presents the conclusion with theoretical and practical implications, and scope for future studies.

## 2 Conceptual framework

### 2.1 Food waste: *Facts and data*

Food waste is defined as ‘*food and the associated inedible parts removed from the human food supply chain in the retail and food service sector as well as households’* [1]. Food loss is the reduction in the quantity or quality of food resulting from decisions and actions by food suppliers in the chain, excluding retail, food service providers, and consumers [5]. Sustainable consumption and production plans to halve the amount of food waste produced per capita at the retail and household levels by 2030 through harvest, production, and supply chains [21, 24, 28, 31]. Approximately one-third (approximately 1.3 billion tons) of human-produced food is wasted or lost as of 2011, and the amount lost at the retail and consumer levels is yet to be accurately estimated [4, 5, 11, 32, 33]. According to national and sectoral reports in the food and supply chain from 2000 to 2017, food loss and waste data at the consumption and household levels, including retail sectors, account for up to 37% [5].

In Korea, solid waste is classified into three categories: recyclables, food waste, and general waste. Food waste collection was stopped in 1996 in landfills that received most of the food waste in Seoul and neighboring districts, and the Ministry of Environment banned direct landfilling for food waste in 2005. The Seoul, Korea's capital city, has faced a huge social issue to dispose of food waste since then [34]. Waste recycling, resources, and circulation have increased public interest [35], and the volume-based waste fee system (VWF) or ‘pay-as-you-throw’ concept was implemented nationwide in Korea [32, 36, 37]. Thus, general and food waste are thrown in standard garbage bags that are produced based on the VWF, while recycled materials are classified into eight types: paper, plastic, glass, cans, scrap, vinyl, clothing, and Styrofoam [38].

Compared to the average annual food waste generation of 95–115 kg per capita in North America and Europe, Koreans produce 130 kg of food waste per capita annually. It has been suggested that this may be due to Koreans’ tradition of enjoying side dishes such as kimchi that are typically disposed of after every meal when left over [39]. However, researchers have opined that there is a lack of statistics on Korean household food waste, according to academics, makes it difficult to make informed policy decisions [24].

Food waste recycling has increased from 2% to 95% [39] and began recycling food waste using special biodegradable plastic bags in 2013 [38,39]. According to the OECD solid waste management data in 2014, Korea has the highest recycling rate (58.1 %) among member countries [36]. Under the Resource Circulation Act 2016 [40], the Korean government set the goal of minimizing landfill and incineration for waste disposal, reducing waste generation to below 20 % in mid-to long-term plans by 2027, increasing recycling rates by 82 %, and reducing final waste disposal from 9 % to 3 % [35].

The Korean government has been piloting ICT-based Radio-Frequency Identification (RFID) devices that can track the collection status of food waste in real time since 2010, but they cover only 25% of the nation [25]. Jeju Island is the smallest province in Korea, with a population of less than 700,000, yet it attracts 10 to 15 million tourists annually [41, 42]. The 628 tons of daily solid waste emissions, including approximately 34% of food waste, is one of the most controversarial social issues on the island [41].

### 2.2 Food waste: *intentional drivers* based on the theory of planned behavior

The theory of planned behavior (TPB) is a modified theory that overcomes the limitation of the aggregation principle in that a single sample of behavior reflects the effects of a variety of different factors unique to a particular situation, occasion, and action observed, which are factors more closely related to that action [43]. The fact that behavioral achievement affects both motivation (intention) and ability (action control) has been applied to many learning theories related to motivation, such as learning and task performance [43, 44].

The flexible research framework of the TPB allows a large number of researchers to extensively design their models to investigate the diverse determinants that support behavioral intentions for reducing food waste in contemporary contexts [17, 45, 46, 47, 48]. Economic factors such as income, price concerns, financial attitudes, food surplus, incentives in logistics, management, administration with corporate support, buying best offers [3, 49, 50], and environmental concerns regarding the knowledge of the amount and separation of food waste [47, 51, 52] are the extended predictors that support the original TPB model for researching food waste.

Sociopsychological factors such as demographic characteristics [7, 48, 53, 54], risk perception [55], habits, emotions, beliefs, personal norms, and moral elements, including feelings of guilt [3, 7, 9, 56], government policies and restrictions [57] provide broader and more diverse determinants to researchers. The solid construction and impact of the theory have been indisputably applied as a theoretical foundation for consolidating the concept of consumer behavior [58] to reduce waste in food waste-related studies since 2013 [9]. However, the TPB does not always support every aspect of the relationship between human behavior and intention [7, 54, 59]. For instance, subjective norms fail to stimulate consumers’ intention to reduce food waste when they are at restaurants [59]. Russell et al. [56] found that attitude does not affect the intention to reduce food waste; rather, negative emotions and habits intervene in stronger behavioral changes, yet as expected, we found that negative emotions were associated with greater intentions to reduce food waste, but contrary to our predictions they were also associated with higher levels of food waste behaviour. In other words, participants who experienced more negative emotion when thinking about food waste intended to reduce their waste but actually ended up wasting more food. Previous research has suggested that reducing food waste from individual behavior requires non-cognitive determinants in addition to great self-control and normative support.

Evans [27] claims that surplus food results from material, cultural, and social conditions in the community where food is wasted, rather than from individual choices, attitudes, and behaviors. Since developed countries experience greater food loss and waste generation [17], financial causes related to the effects of individual behaviors on food consumption should be considered in the extended research model [3, 5, 20, 59, 60, 61, 62, 63, 64]. Increasing income affects food culture consumption patterns for each generation, resulting in a variety of studies based on age, education, employment status, family size, and number of children; therefore, these factors should be considered [5, 6, 48, 62, 64].

In addition to above mentioned variables, pathological factors derived by COVID-19 pandemic have changed people's food consumption habits. Many countries no longer allow the usual meeting, eating, drinking, and social contact with people they previously considered routine due to the unprecedented outbreak of COVID-19 since the end of 2019 [23,64]. Food consumers panic buy and hoard food, and mobility restrictions have significantly reduced the number of people meeting or eating out. This worrying situation continues in cities and countries experiencing lockdowns.

Although COVID-19 has spread across the country, there has been no nationwide lockdown in South Korea since the end of 2019. However, as the number of confirmed cases increases day by day, the number of unemployed in the lodging and food service sectors has raised considerably [65]. However, the consumption pattern of Koreans, which has slowed down due to a surge in coronavirus cases, has returned to normal as the situation returns to normal and is now showing signs of avoiding direct social contact by using online purchasing or delivering food services [66]. Therefore, the COVID-19 pandemic has become a common indicator of people’s risk concerns and precautions.

In this study, we implemented the modified TPB model as an indicator of local residents’ intention to reduce food waste, including consumer price consciousness and risk concerns due to COVID-19, and socio-demographic factors as antecedent variables that affect individual behavioral intentions in research related to food waste. The detailed hypotheses based on the conceptual model are discussed in the next section.

## 3 Materials and methods

### 3.1 Research model and hypotheses: study 1

**Figure 1:**
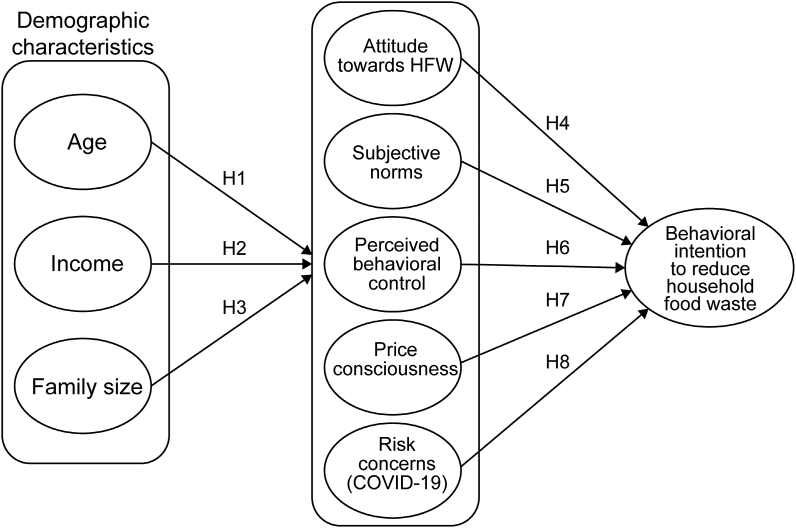
Conceptual model for behavioral intention of food waste reduction intentions.

To identify factors that affect the degree of food waste reduction behavior of general residents in South Korea, including Jeju Province, this study aimed to analyze data through a structural equation model applied with the partial least squares method for testing the influence of multiple determinants and parameters on dependent variables. In the basic concept of TPB, the predictors used as dependents become mediators, socio-demographic factors were assumed as independent variables, and the intention to reduce household food waste was defined as the dependent variable in this model. This study initially determined the relationships between demographic factors and two vulnerable factors: economic factors highly dominated by the consumer price index and the impact of COVID-19 as an emerging disaster. The study’s conceptual framework is shown in Figure 1, and the hypotheses of the study to be tested are as follows:

Hypothesis 1 ***(H1)*** Age affects behavioral intentions to reduce household food waste.

Hypothesis 2 ***(H2)*** Income affects behavioral intentions to reduce household food waste.

Hypothesis 3 ***(H3)*** Family size affects behavioral intentions to reduce household food waste**.**

Hypothesis 4 ***(H4)*** Attitudes toward household food waste affect behavioral intentions to reduce household food waste.

Hypothesis 5 ***(H5)*** Subjective norms on reducing household food waste affect behavioral intentions to reduce household food waste.

Hypothesis 6 ***(H6)*** Perceived behavioral control over reducing household food waste affects behavioral intentions to reduce household food waste*.*

Hypothesis 7 ***(H7)*** Food price consciousness affects behavioral intentions to reduce household food waste.

Hypothesis 8 ***(H8)*** Risk concerns due to COVID-19 affect behavioral intentions to reduce household food waste.

### 3.2 Hypotheses: study 2

This study was completed by clarifying the differences in residents’ perceptions of household food waste in Korea due to regional boundaries. Although the amount of regional physical food waste can be measured locally from waste collection companies or RFID devices, it is difficult for researchers to determine residents’ perceptions because there is no unified measurement tool available to analyze them as data from the devices. Thus, the researchers conducted an advanced analysis using the multi-group analysis (MGA) method of the Partial Least Squares Structural Equation Modeling (PLS-SEM) based on the conceptual research model presented above.

Hypothesis 9 ***(H9)*** There are statistically significant differences in the effect of socio-demographic factors on the parameters affecting the intention to reduce household food waste between the residents of Jeju Province and mainland Korea.

**Figure 2:**
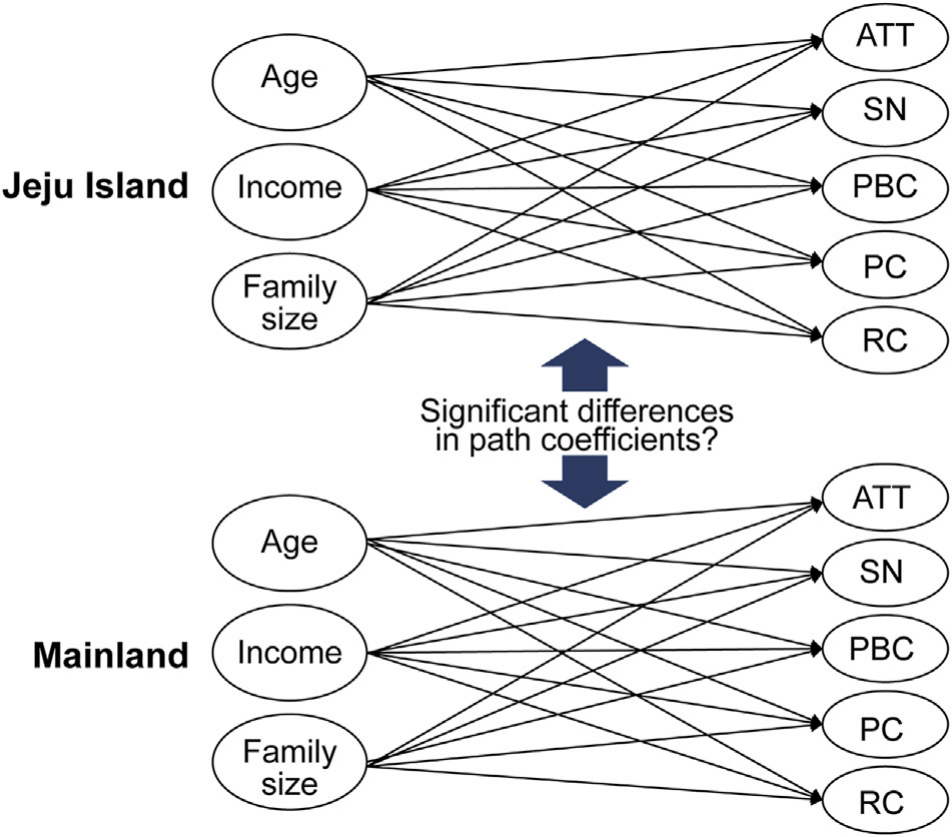
Research model for the ninth hypothesis using multi-group analysis in Partial Least Squares Structural Equation Modeling.

By comparing the path coefficients exhibiting differences in perceptions between residents of Jeju Province and other provinces in the Korean mainland, practical implications can be drawn based on the results. For this analysis, researchers examined the paths where the cognitive difference between the two groups was statistically significant among the three socio-demographic factors and the five factors used as parameters (a total of 15 paths) by applying the model of **Study 1**. An additional research model for this hypothesis is shown in Figure 2.

### 3.3 Samples, data collection, and analyses

With a population of 51,672,400, South Korea had 1,273,766 cumulative confirmed cases of COVID-19 on June 30,2021, and 282 deaths. Meanwhile, the Jeju Special Self-Governing Province has a population of 675,293. Since the first confirmed cases of COVID-19 were reported in February 2020, the cumulative number of confirmed COVID-19 cases was 1,262 and 1 death, and the average number of daily confirmed cases at 8.7 over the two weeks during the survey period [67, 68].

In this study, we used IBM SPSS 24.0 (IBM Corp. Released 2016. IBM SPSS Statistics for Windows, Version 24.0. Armonk, NY: IBM Corp. Source: https://www-01.ibm.com/support/docview.wss?uid=swg21476197), to conduct a basic frequency and factor analysis to identify the general socio-demographic characteristics of respondents, and SmartPLS 3.0 (Henseler et al. [69]: Smartpls 3. Bönningstedt: SmartPLS. Retrieved from http://www.smartpls.com. SmartPLS GmbH, Oststeinbek, Germany), for a path analysis to determine the reliability, validity, model fit, and coefficients, including total and specific indirect variables.

Respondents who participated in the survey indicated the degree to which they agreed with their subjective opinions in a self-marking-style mobile survey form for two to five questions presented for each factor. The three main independent variables (attitude, subjective norm, and perceived behavior control) that affect behavioral intention in the TPB, and price consciousness and risk concerns due to COVID-19, which were adopted as the main variables in this study, were presented excluding the question of final intention to reduce food waste. Participants marked items that fit the Likert 5 scale according to their views on the question, for a total of 5° from ‘strong negative/disagree’ to ‘moderate/neutral’ and ‘strongly positive/agree.’

All data collection was connected to the mobile and online link sites, and it was impossible to proceed to the next question if there was an unanswered question; hence, there was no non-response data. However, only 508 cases were used for the analysis because 5 out of 513 individual data were excluded from the analysis due to uncertain reliability. Among each latent variable group, one item from each attitude (ATT 5), perceived behavioral control (PBC1), and price consciousness (PC5) were eliminated because of low reliability/outer loadings.

Considering the reliability of each factor, using IBM SPSS (International Business Machines Corporation (IBM), New York, United States), it was confirmed that the Cronbach alpha value was 0.7 or higher, which was also confirmed in the second test using Smart-PLS. The demographic characteristics investigated in this study were gender, age, educational background, monthly income, occupation, number of families, and regional variables.

The need for ethical approval was waived by the XXX [Blinded for Review] University’s review board as the research article collected no sensitive personal information. In the process of data collection, informed consent was obtained by individuals on an online form.

## 4 Results

### 4.1 Frequency analysis and model assessment

The socio-demographic characteristics of the participants are presented in Table 1. The male to female ratio of the respondents was 47.8:52.2. The age group was relatively evenly distributed, with those in their 50s accounting for 24.4% (the largest group), followed by those in their 40s, 20s, and 30s, and those in their 60s (40%, 20%, 30%, and 16.7%, respectively). University undergraduates accounted for the largest proportion (53.5%), and the educational background of the respondents was distributed in the order of high school, graduate school, and college. The highest monthly income groups ranged from less than US$ 1,000 and US$ 2,000 to US$ 2,999, with 13.2% of respondents earning more than US$ 5,000 per month. Among the participants, white-collar workers accounted for 28.3%, followed by those belonging to other occupational groups (15.6%), followed by housewives and students (14.4% and 13.8%, respectively). Of the participants, 30.9% had three-person households, 27.8% had four-person families, 17.7% were single or two-person households with the same ratio, and 5.9% were large households with five or more members. During the survey period (from June 2021 to July 2021), when the research was conducted, the population of Jeju Province was 675,293, and the total population of 16 other metropolitan cities and provinces in South Korea was 50,997,107. Accordingly, the total population of the Republic of Korea is 51,672,400. Jeju Province is a self-governing province that accounts for approximately 1.30% of the nation’s population with a unique isolated environment away from the mainland.

**Table 1:** Socio-demographic characteristics of samples.

Particular		n	%	Particular		n	%
Gender	Male	243	47.8	Occupation	White-collar	144	28.3
	Female	265	52.2		Professional	49	9.6
Age	20s	94	18.5		Mechanics	30	5.9
	30s	92	18.1		Service sector	29	5.7
	40s	113	22.2		Self-employed	33	6.5
	50s	124	24.4		Housewife	73	14.4
	60s & above	85	16.7		Student	70	13.8
Educational Level	High school	88	17.3		Agriculture/Fishery	1	0.2
	College	67	13.2		Others	79	15.6
	Undergraduate	272	53.5	Family Size	1	90	17.7
	Graduate school	81	15.9		2	90	17.7
Monthly Income	Below $ 1,000	122	24.0		3	157	30.9
	$ 1,000–$ 1,999	63	12.4		4	141	27.8
	$ 2,000–$ 2,999	121	23.8		5 or more	30	5.9
	$ 3,000–$ 3,999	85	16.7	Region	Jeju Island (Province)	208	40.9
	$ 4,000–$ 4,999	50	9.8		Mainland	300	59.1
	$ 5,000 & above	67	13.2	**Total**		**508**	**100.0**

Table 2 shows the validity and reliability assessment results for each measured variable constituting the latent variables of this research model. Three items in risk concerns due to COVID-19 were unstable, so we decided to remove them using two items with the highest loadings. In particular, two items in each variable, except for risk concerns (RC) and behavioral intention (BI), had relatively lower loadings, resulting in less than 0.50 reliability. However, all four items made above-average combinations to exhibit moderate convergent validity and overall internal consistency reliability.

**Table 2:** PLS-SEM assessment results of measurement models.

Latent Variable	Indicator	Convergent Validity			Internal Consistency Reliability			
		Outer Loadings	Reliability of variables	AVE	Cronbach’s Alpha		rho_A	Composite Reliability
**Attitude (ATT)**	ATT1	0.663	0.440	0.832		0.734	0.777	0.557
	ATT2	0.821	0.674					
	ATT3	0.846	0.716					
	ATT4	0.632	0.399					
**Subjective Norms (SN)**	SN1	0.876	0.767	0.875		0.714	0.715	0.778
	SN2	0.888	0.789					
**Perceived Behavioral Control (PBC)**	PBC2	0.794	0.630	0.832		0.733	0.752	0.555
	PBC3	0.680	0.462					
	PBC4	0.814	0.663					
	PBC5	0.682	0.465					
**Price Consciousness (PC)**	PC1	0.789	0.623	0.877		0.814	0.818	0.640
	PC2	0.808	0.653					
	PC3	0.812	0.659					
	PC4	0.792	0.627					
**Risk Concerns due to COVID-19 (RC)**	RC1	0.812	0.659	0.867		0.709	0.828	0.765
	RC2	0.934	0.872					
**Behavioral Intentionto Reduce Household Food Waste (BI)**	BI1	0.771	0.594	0.890		0.845	0.847	0.618
	BI2	0.796	0.634					
	BI3	0.820	0.672					
	BI4	0.788	0.621					
	BI5	0.753	0.567					

Variance inflation factors (VIF) is an indicator that provides a measure of the number of times the target variable is larger for multicollinear data than for orthogonal data. A VIF value above 5 indicates that there is a potential problem in the inner structure [70]. Multicollinearity was not observed in this model since all VIF values were below 2. Meanwhile, the Heterotrait-Monotrait ratio of correlations (HTMT) measures the discriminant validity in partial least equation modeling, a method more stringent than the cross-loadings or the Fornell-Larcker criterion [69]. Researchers applied HTMT as suggested by Hair et al. [71], based on the threshold values between 0.85 and 0.90. All HTMT values are lower than 0.75, and the bootstrapping results show that 1 is not contained within the interval range (based on 95% confidence level); thus, discriminant validity was also established.

### 4.2 Research test (path analysis): study 1

As described in the previous section, three socio-demographic factors were considered as the antecedent/independent variables for the path analysis model in the study after attempting every factor of the demographic characteristics. This indicates that all other factors, such as gender, academic background, and occupation, did not affect the model. As seen in Table 3, all three independent variables have a certain effect on their mediators (H1, H2, and H3), supporting our hypothesis that socio-demographic factors affect the behavioral intention of residents to reduce food waste. The direct effects should be considered (see Table 3); however, we found that age affects all mediators (p < 0.001, t = 2.906 to 6.845), while the effectiveness of income and family size was limited to certain determinants. For example, income variation can be explained only by risk concerns (p < 0.000, t = 5.278), and variations in residents’ family size affect only subjective norms (p < 0.01, t = 2.722) and perceived behavioral control (p < 0.01, t = 2.621). The three mediators, attitude, subjective norms, and perceived behavioral control from the original TPB model support our hypotheses significantly (p < 0.001, t = 2.660 to 11.868) and price consciousness (p < 0.000, t = 3.529), and the fourth factor in the model. Only risk concerns do not support these hypotheses.

**Table 3:** Research hypotheses test (path analysis).

Hypotheses (Path)		f^2^	Path coefficient	t-statistics	p-value	95% BCa confidence interval	Results
H1	Age → ATT	0.017	1.148	*3.026*	*0.003*	[0.042, 0.227]	Supported
	Age → SN	0.023	1.148	***3.568***	***0.000***	[0.064, 0.236]	Supported
	Age → PBC	0.027	1.148	***3.801***	***0.000***	[0.079, 0.253]	Supported
	Age → PC	0.015	1.148	***2.906***	***0.004***	[0.034, 0.214]	Supported
	**Age → RC**	0.093	1.148	***6.845***	***0.000***	[-0.395, -0.221]	Supported
**H2**	Income → ATT	0.000	1.163	0.459	0.641	[-0.049, 0.117]	Not supported
	Income → SN	0.004	1.163	1.493	0.120	[0.035, 0.208]	Not supported
	Income → PBC	0.003	1.163	1.266	0.206	[-0.187, -0.028]	Not supported
	Income → PC	0.006	1.163	1.786	0.083	[-0.056, 0.132]	Not supported
	**Income → RC**	0.046	1.163	***5.278***	***0.000***	[-0.031, 0.152]	Supported
**H3**	Family Size → ATT	0.001	1.019	0.743	0.451	[-0.118, 0.079]	Not supported
	**Family Size → SN**	0.016	1.019	***2.722***	***0.007***	[-0.024, 0.148]	Supported
	Family Size → PBC	0.012	1.019	***2.690***	***0.010***	[-0.031, 0.141]	Supported
	Family Size → PC	0.001	1.019	0.781	0.405	[-0.168, 0.006]	Not supported
	Family Size → RC	0.004	1.019	1.288	0.207	[0.131, 0.297]	Not supported
**H4**	ATT → BI	0.175	1.365	***7.730***	***0.000***	[0.239, 0.401]	Supported
**H5**	SN → BI	0.016	1.295	***2.660***	***0.009***	[0.029, 0.162]	Supported
**H6**	**PBC → BI**	0.393	1.272	***11.868***	***0.000***	[0.385, 0.532]	Supported
**H7**	PC → BI	0.037	1.139	***3.529***	***0.000***	[0.064, 0.209]	Supported
**H8**	RC → BI	0.007	1.038	1.733	0.096	[-0.121, 0.001]	Not supported

**Overall fit of the Estimated Model**	**Value**	**Endogenous variable**	**R**^**2**^	**R**^**2**^**Adj.**	**Q**^**2**^
SRMR	0.065	Attitude Subjective Norms	0.018	0.012	0.006
			0.052	0.047	0.037
d_ULS	1.262	Perceived Behavioral Control	0.051	0.045	0.024
d_G	0.471	Price Consciousness	0.017	0.011	0.008
X^2^	1439.137	Risk Concerns	0.103	0.098	0.072
NFI	0.683	Behavioral Intention to Reduce HFW	0.576	0.572	0.347

∗∗∗p < 0.001, ∗∗p < 0.01, ∗p < 0.05.

Assessing model fit in PLS-SEM is primarily based on predictive efficiencies, unlike Covariance-Based Structural Equation Modeling, which indicates the suitability of the interpretation of the research model for explaining the tests of the hypotheses depending on the path coefficients, the level of the R^2^ values, and the effect size *f*^*2*^ [71] in Table 3. Choosing the estimated model when summarizing the overall model fit is recommended, as it remains arguable owing to its early phase of advancement. Standardized root mean square residual (SRMR) values below 0.10 or 0.08 are considered a good fit, while NFI, the Chi^2^ value of the model at 1 divided by the Chi^2^ value of the null model that is closer to 1 is considered good for model fit. As a result, the NFI produces values between 0 and 1. The closer the NFI is to 1, the better is the fit [71].

The coefficient of R^2^ determination that provides the explanatory power of prediction of the model is usually considered high at 0.26 or more, moderate at 0.13–0.26, and low at below 0.13 [72]. The explanatory coefficient (R^2^) for behavioral intention to reduce HFW, the dependent variable of this study, is considered very high at 0.576, and the adjusted R^2^ coefficient reaches 0.572, indicating that the slight difference does not affect the model construction.

The results of the overall and specific indirect effects of all predictors in the model showed that age had very strong indirect effects (p = 0.000, t = 4.862) on behavioral intention. However, the path from age to behavioral intention mediated by price consciousness and risk concerns was insignificant (p = 0.051, t = 1.644 and p = 0.106, t = 1.620), showing no mediating effects of those paths in this case. All paths from income to behavioral intention also had no indirect effects. However, family size is mediated by perceived behavioral control, which indicates that it has indirect effects in the model with p < 0.01 and t = 2.673. Finally, the researchers have drawn out the structural modeling of the path analysis, capturing age as the most effective predictor among socio-demographic factors and both subjective norms and perceived behavioral control as the most effective mediators in the local context for reducing household food waste.

**Figure 3:**
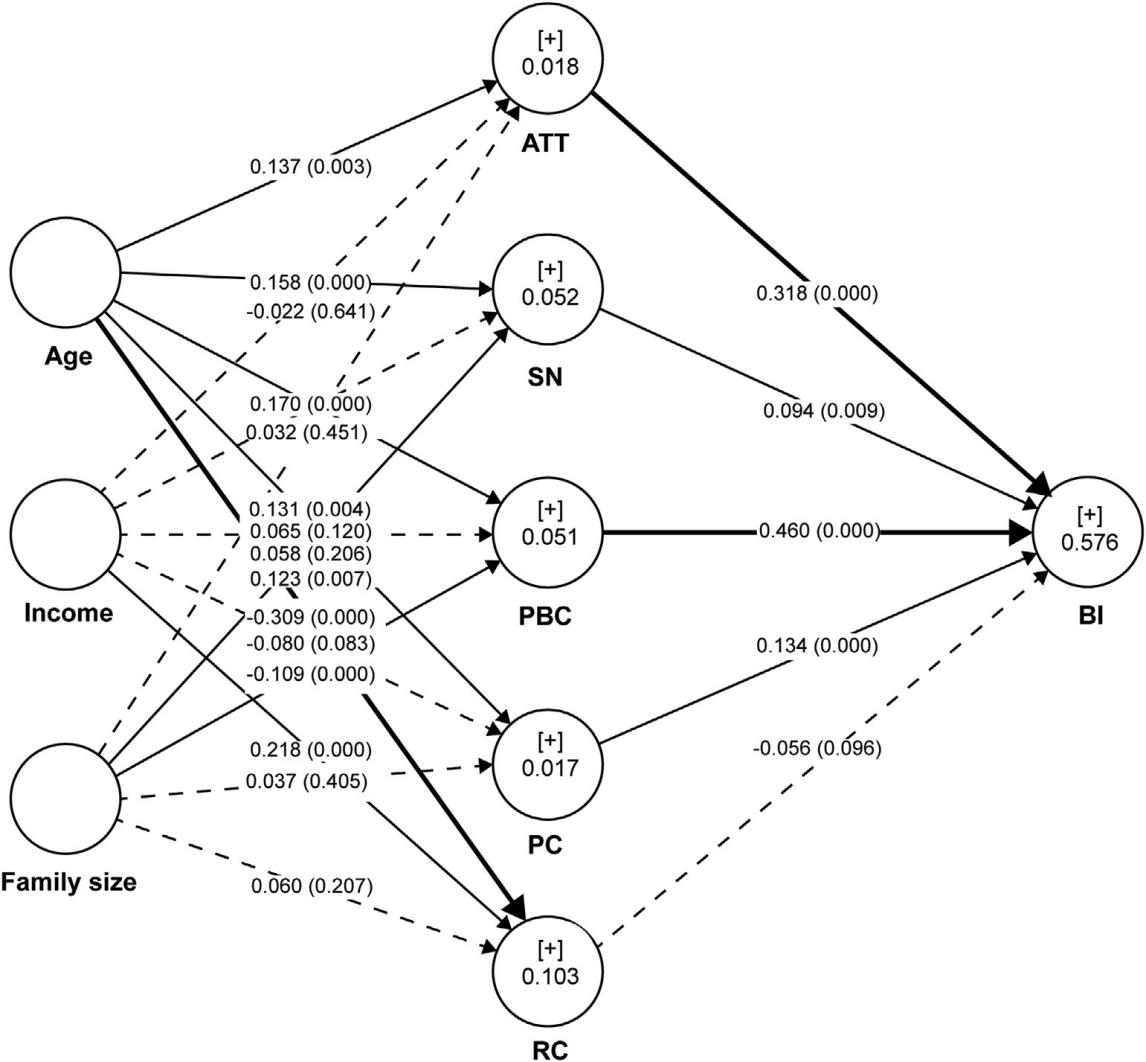
Path Analysis results of PLS-SEM for behavioral intention of household food waste in Korea.

Figure 3 shows the most influential variables for each path for the eight hypotheses of **Study 1** raised in this paper. The solid line indicates significant influence, and the dotted line indicates an insignificant influence. The variable showing the most effective path among the demographic antecedents in the research investigated during the period in which COVID-19 was in the sphere of influence was age, and it was established that age and risk concerns about COVID-19 were directly proportional. However, it was found that risk concerns due to COVID-19 did not affect the intention to reduce food waste. Among the parameters, the variables that have the highest influence on the intention to reduce food waste are perceived behavioral control and attitude. In particular, perceived behavioral control was found to have the strongest effect on intention, as it was also affected by age and number of household members.

As the research was conducted in both Jeju Province and the Korean mainland, the second part of the research assessed the extent to which the differences between the residents in different areas were significant, using a multi-group analysis (MGA).

### 4.3 Research test (multi-group analysis): study 2

The minimum required size of the full dataset in this study was 222 when assuming a moderate effect size (f^2^ **=** 0.15). The actual and effective sample size for the full dataset was 508, which was sufficiently large to avoid sampling errors. The researchers examined two structural models with separate datasets using categorical variables. The regional barrier was the only variable we considered before conducting the MGA since Jeju is an isolated self-governing province in Korea. Table 4 displays the results of path coefficient (R^2^), predictive relevance (Q^2^), and effect sizes (f^2^) of the datasets.

**Table 4:** Results of path coefficient (R^2^), predictive relevance (Q^2^), and effect sizes (f^2^).

	Jeju Province			Other Provinces			Full Data		
	R^2^	Q^2^	f^2^	R^2^	Q^2^	f^2^	R^2^	Q^2^	f^2^
ATT	0.049	0.035	0.205	0.030	0.021	0.166	0.018	0.006	0.175
SN	0.077	0.063	0.011	0.075	0.066	0.031	0.052	0.037	0.016
PBC	0.061	0.047	***0.312***	0.054	0.045	***0.520***	0.051	0.024	***0.393***
PC	0.049	0.035	0.041	0.007	-0.003	0.038	0.017	0.008	0.037
RC	0.171	0.159	0.000	0.093	0.084	0.025	0.103	0.072	0.007
BI	***0.530***	***0.518***	-	***0.656***	***0.650***	-	***0.576***	***0.347***	-

The path coefficient R^2^ values are the result of endogenous constructs. The R^2^ values of the dependent variable (BI) in the three models were 0.530, 0.656, and 0.576, respectively, when comparing Jeju Province (n = 208), other provinces (n = 300), and the full dataset (n = 508). The Q^2^ values indicate the combined features of in-sample explanatory power and out-of-sample predictions [73]. The Q^2^ values in all cases showed medium predictive relevance of 0.518, 0.650, and 0.347, respectively. The f^2^ values represent the rank of the relevance predictors in the structural model. In this study, we found that PBC plays a major role in behavioral intentions. The effect sizes of PBC in the Jeju Province model (f^2^ = 0.312) was higher than recommended, followed by the full data set (f^2^ = 0.520) and other province models (f^2^ = 0.393), which was higher than the medium effect size.

The final step of the MGA examined whether there were differences between the two regional groups and their path coefficients. The purpose of **Study 2** was to examine the effects of socio-demographic factors between the island and the Korean mainland. Statistical significance was tested using the newly calculated p-values, as shown in Table 5. Three paths differed significantly between Jeju Province and the other provinces: age and PC (p < 0.05, t = 2.519), age and RC (p ≤ 0.001, t = 3.686), and family size and RC (p < 0.05, t = -2.683).

**Table 5:** Results of Multi-group analysis (MGA).

	Path Coefficients- diff (Jeju - Others)	t-statistics (Jeju vs Others)	p-Value (Jeju vs Others)	p-Value New(Jeju vs Others)
Age → ATT	0.105	1.372	0.113	0.226
Age → SN	0.058	-0.075	0.271	0.541
Age → PBC	0.050	-0.268	0.303	0.605
**Age → PC**	0.176	***2.519***	0.024	***0.048****∗*
**Age → RC**	-0.276	***3.686***	0.999	***0.001****∗∗*
Income → ATT	-0.141	-0.226	0.919	0.162
Income → SN	-0.051	-1.289	0.706	0.588
Income → PBC	-0.137	-1.797	0.914	0.171
Income → PC	-0.161	2.371	0.959	0.082
Income → RC	0.148	1.938	0.043	0.086
Family Size → ATT	0.018	0.125	0.418	0.837
Family Size → SN	-0.025	-0.676	0.614	0.772
Family Size → PBC	-0.027	0.074	0.626	0.748
Family Size → PC	-0.018	-0.341	0.576	0.847
**Family Size → RC**	-0.216	***-2.683***	0.992	***0.016****∗*

∗∗∗p < 0.001, ∗∗p < 0.01, ∗p < 0.05.

## 5 Discussion and conclusions

This study established the predictors that could be applied to residents’ attitude toward reducing food waste in Jeju Province, Korea’s leading tourist destination, and the mainland of Korea. Based on Azjen’s TPB, numerous researchers have applied several predictors to fit the research model of structural equations using the partial least equation. We evaluated the measurement variables for the model fit before analyzing the effect of each variable through path analysis for total 508 data responded to the mobile survey.

Socio-demographic factors are predictors that many researchers in food waste research have applied to modified TPB models to present their results and implications. Similar to the findings of Aschemann-Witzel et al. [74], Li et al. [6], Visschers et al. [7], and van der Werf et al. [54], we identified age as the most influential antecedent, in a positive direction, for behavioral intention of household food waste. The factor most affected by age was risk concerns with the mean value drawn as negative (M = -3.09, p = 0.000), suggesting that senior citizens may be consuming less delivery food, even during the COVID-19 pandemic, and usually eat out less than younger people. Overall, individuals’ normative beliefs and attitudes toward reducing food waste are directly and positively involved in determining their behavioral intentions as they age.

The effectiveness of income factors works in the same way as age factors in the opposite direction. Individuals who earn more eat out more when there is no quarantine risk and consume more delivery food while maintaining social distance. However, generating household food waste does not always depend on income. Some lower-income households do not produce food waste compared to those with higher incomes [3, 58]. This implies that there are complex determinants that control the perceptions or behavioral intention to reduce food waste in households [75].

In addition, it was established that family size does not directly affect behavioral intention in reducing household food waste; however, it is significant when mediated by perceived behavioral control in our model.

Overall, individuals’ incomes affected the risk concerns due to COVID-19. Risk concerns in this research are precisely about the changes in food consumption patterns over COVID-19, so that the respondents’ habits could be assumed from their answers. Our results suggest that compared to respondents with lower incomes, respondents with higher incomes used to eat out more before COVID-19 and tend to consume more home delivery food during the COVID-19 pandemic; however, this does not affect the behavioral intention to reduce household food waste.

We believe that it is necessary to think about the research method of the literature that has produced a positive correlation that households with higher incomes produce more food waste [3, 58] or vice versa [6], among the many prior studies that clearly show a relationship between income and food waste emissions. When people are concerned about household food waste, we generally think of food waste generated before and after cooking. However, considering food waste generated by delivery or takeout food during a pandemic situation such as COVID-19, it is important to measure and track them in both food waste and plastic containers, which are unnecessarily generated waste.

Family size affects only perceived behavioral control in our model, which indicates that it is not as effective as many previous studies in other countries that concluded that larger family sizes generate more food waste [6, 7, 62]. However, perceived behavioral control, including self-control to buy, cook, and waste less amount of food and family members’ support in bigger families of households did not influence the intention to reduce household food waste. The total indirect effects of family size on behavioral intention through perceived behavioral control (β = -0.050, p = 0.008) proved that respondents in smaller families have higher self-control perception and intention to reduce food waste, or they may experience more psychological burden. The results of the total indirect effects of PLS-SEM are useful for ensuring the mediating effects of variables in structural equation models.

Second, the results of this study confirm the significance of the TPB in this research model. Visschers et al. [7], Graham-Rowe et al. [9], Aktas et al. [49], and Stefan et al. [58] demonstrated the primary role of three independent variables (attitude, subjective norms, and perceived behavior controls) and personal norms and moral norms/attitudes in extended TPB models for reducing food waste. Particularly, in this study, socio-demographic variables were identified as effective predictors, and among the three variables above, perceived behavior controls were identified as those most affected by antecedent variables. Age, in particular, appeared to have the greatest impact on individual norms, confirming that it is in line with what was presented in the first conclusion.

Finally, the mediators considered that the concept of an extended model in this paper entailed price consciousness and risk concerns from COVID-19. In conclusion, price consciousness influenced residents’ intentions to reduce household food waste, while risk concerns caused by COVID-19 were not statistically significant. Interestingly, the price consciousness of consuming food is also closely connected with food taste in the restaurant context of customers’ food waste behavior [59]. The direct effects of all socio-demographic variables are statistically significant to a specific extent, as published by the FAO [5]; the amount of food waste emissions is often proportional to the family size in most case studies [6, 7, 62, 64, 76].

It can be interpreted that the effects of COVID-19 in Korea change people’s propensity to eat out, deliver food, and online grocery buying patterns, but are not as strong as in other countries or cities where people experience lockdown, such as Italy [31] and Spain [23]. People in these countries and cities have shown strong intentions and awareness of food waste and their availability during in an economic crisis. This suggests that it is closely related to COVID-19 transmission in the region. Fortunately, Jeju Island is exposed to the risk of influx of tourists amidst the COVID-19 pandemic, but COVID-19 did not have a significant impact on the generation of household waste. Unlike in Jeju Province, however, risk concerns due to COVID-19 differ in other Korean provinces. The results of the multi-group analysis indicated that risk concerns caused by age and family size differed significantly between the two regions.

As previously suggested in this paper, the number of confirmed cases of COVID-19 in Jeju Province only exceeded 1,200 in the past 17 months. Conversely, as of July 13 in 2021, the number of confirmed cases in the province was only 1,352, accounting for only 0.85 % of the 159,655 confirmed cases in Korea [67]. However, the situation is different considering demographics. The proportion of confirmed cases in Jeju Province accounts for 0.19% of the province’s total population of approximately 690,000. This is similar to Busan, which is Korea’s second-largest metropolitan city with a population of nearly 3.5 million. This is understandable considering the fact that Jeju Province has a potentially complicated factor that only considers the resident population due to the influx of tourists [42]. Jeju Province is a tourist destination amid all the economic, social, and environmental phenomena and interests, and seeking cooperation and understanding from residents in all policies and administrative affairs is indispensable.

Through this study, we have attempted to draw people’s attention to the food waste problem caused by the altered food consumption patterns during the COVID-19 pandemic. Although we tried to compare Jeju, a tourists island destination, with the most densely populated areas, we admit that the number of data used in this study is insufficient to claim generalization. Therefore, we acknowledge the need to continue with follow-up studies to confirm whether there was a change in people’s perceptions before and after the pandemic by actively expanding the sample in similar studies after the pandemic.

## Declarations

### Author contribution statement

Mona Chang: Contributed reagents, materials, analysis tools or data; Wrote the paper.

Walimuni Arachchilage C. S. M.: Conceived and designed the experiments; Performed the experiments; Analyzed and interpreted the data.

Wisurumuni Arachchilage Hasitha Maduranga Karunarathne: Performed the experiments.

Min-cheol Kim: Conceived and designed the experiments; Analyzed and interpreted the data; Wrote the paper.

### Funding statement

This research did not receive any specific grant from funding agencies in the public, commercial, or not-for-profit sectors.

### Data availability statement

Data will be made available on request.

### Declaration of interest’s statement

The authors declare no conflict of interest.

### Additional information

No additional information is available for this paper.
