# Supplementary material for: Residents’ perceptions of household food waste during the COVID-19 outbreak in Korea
Source: Heliyon. 2022 Nov 11;8(11):e11439. doi: 10.1016/j.heliyon.2022.e11439 (PMC9650487; doi:10.1016/j.heliyon.2022.e11439)
Supplement: Appendix [file mmc1.docx]

**Appendix.** Survey questionnaire items.

**Attitudes (ATT)**

• It is unnecessary to waste food: it can always be used in some way.

• It upsets me when unused products end up in the waste bin or garburator.

• I think that wasting food is a waste of money.

• Throwing out food does not have an environmental impact.

• I am not worried that eating leftovers results in health damage (*eliminated*).

**Subjective Norms (SN)**

• People who are important to me find my attempts to reduce the amount of food wasted unnecessary.

• People who are important to me disagree when I try to reduce food waste.

**Perceived Behavioral Control (PBC)**

• I find it easy to prepare a new meal from leftovers (*eliminated*).

• I find it easy to ensure that only small amounts of food are discarded from my household.

• I find it easy to plan my food shopping in such a way that all the food I purchase is eaten.

• I feel that I can do something about the food wasted in my household.

• Other household members make it impossible for me to reduce the amount of food wasted in my household.

**Price Consciousness (PC)**

• I buy less agricultural food stuff because of its price incensement.

• I buy less livestock food stuff because of its price incensement.

• It is important to me that I obtain quality for money.

• I always try to obtain the best quality at the best price.

• Special offers in supermarkets make me buy more food than necessary (*eliminated*).

**Risk Concerns (COVID-19) (RC)**

• I think I throw more food waste at home during COVID-19, since I go out eating less.

• I think I throw more food waste at home during COVID-19, since I consume more home-delivered food.

• I buy more products to reduce the activities related to the buying process during COVID-19 (*eliminated*).

• I think that consuming leftovers is harmless considering the seriousness of the COVID-19 crisis (*eliminated*).

• I mostly purchase food online to avoid contamination with COVID-19 (*eliminated*).

**Behavioral Intentions (BI)**

• I try not to waste food at all.

• I always try to eat all purchased foods.

• I try to produce very little food waste.

• I aim to use all leftovers.

• I have a very strong intention to reduce food waste in my home in the following week.
